# Baseline Predictors of Study Discontinuation in a Preclinical Alzheimer’s Disease Clinical Trial

**DOI:** 10.1002/alz.093124

**Published:** 2025-01-09

**Authors:** Rema Raman, Kedir Hussen, Michael C. Donohue, Oliver Langford, Doris P. Molina‐Henry, Paul S. S. Aisen, Reisa A Sperling, Joshua D Grill, the A4 Study Team

**Affiliations:** ^1^ Alzheimer’s Therapeutic Research Institute, University of Southern California, San Diego, CA USA; ^2^ Center for Alzheimer’s Research and Treatment, Department of Neurology, Brigham and Women’s Hospital, Harvard Medical School, Boston, MA USA; ^3^ Institute for Memory Impairments and Neurological Disorders, University of California, Irvine, Irvine, CA USA

## Abstract

**Background:**

Participant dropout from study treatment in a clinical trial can leave a trial underpowered, produce bias in statistical analysis, and limit interpretability of study results. Retaining participants in clinical trials for the full study duration is therefore as important as participant recruitment. This analysis aims to identify the baseline characteristics of participants who discontinued during the blinded phase of one of the first and largest preclinical AD trial completed to date, the Anti‐Amyloid treatment in Asymptomatic AD (A4) Study.

**Method:**

The sample consisted of all A4 trial randomized participants followed for 4.5 years through the COVID‐19 pandemic. Baseline demographic (with age centered at 73 years), clinical, amyloid PET and genetic predictors of study discontinuation were evaluated using a univariate generalized linear mixed model (GLMM), with study discontinuation status as the binary outcome, each predictor as a fixed effect, and site as a random effect to account for differences among study sites in the trial. Characteristics significant at p<0.10 were then included in a multivariable GLMM.

**Result:**

Of 1169 randomized participants, 339 (29%) discontinued from the trial (median follow‐up time: 759 days). Main reasons included participant unwilling to continue (55.8%) and adverse events (18.9%). From the multivariable analysis, the two main predictors of study discontinuation were baseline State‐Trait Anxiety Inventory (STAI) scores (OR = 1.07 [95%CI = 1.02; 1.12]; p = 0.0024) and age (OR = 1.06 [95%CI = 1.03; 1.09]; p<0.0001). Participants with a family history of dementia (OR = 0.75 [95%CI = 0.55; 1.01]; p = 0.0627) and APOEe4 carriers (OR = 0.79 [95%CI = 0.6; 1.04]; p = 0.0941) were less likely to discontinue from the study, with the association being marginally significant. In these analyses, sex, race and ethnic underrepresented group (combined due to small sample sizes), cognitive scores and amyloid/tau PET scores were not associated with study dropout.

**Conclusion:**

Older participants and those with higher levels of anxiety at baseline were more likely to discontinue while those who had a family history of dementia or were APOEe4 carriers were less likely to dropout. Continued analyses aim to identify post‐baseline and longitudinal predictors of attrition in this preclinical AD trial.

